# A foundation’s view of WHO’s urban health research agenda

**DOI:** 10.2471/BLT.23.290135

**Published:** 2023-08-01

**Authors:** Susanna Hausmann-Muela

**Affiliations:** a Fondation Botnar, St Alban-Vorstadt 56, 4052 Basel, Switzerland.

In his opening speech of the World Health Summit in October 2022, the World Health Organization (WHO) Director-General called on all countries to make the needed “paradigm shift, recognizing that health starts not in hospitals and clinics, but in homes, streets, schools and workplaces.”^1^

To support this paradigm shift, WHO has developed an Urban Health Research Agenda that sets the research priorities for addressing the needs and opportunities of urban dwellers.^2^ WHO took an inclusive, multisectoral approach in developing this agenda by holding a consultative process with diverse stakeholders, including governments, civil society, funding bodies, as well as actors from outside the health sector such as urban planning, transport and housing. 

The agenda promotes multisectorial and multistakeholder processes for addressing determinants and drivers of health. The underlying philosophy of the agenda is framed in a set of guiding principles, including adopting a systems approach, co-producing knowledge, and applying an equity and sustainability lens to inform and steer research and the implementation of initiatives. Applying these principles stimulates profound reflections on the approaches to improving the health and well-being of urban dwellers, leading to a shift in focus from the what to the how; that is, to new modes and mechanisms to make cities more conducive environments to healthy societies. 

The flexibility and agility of philanthropic foundations can add value to advancing the urban health research agenda by testing new grounds in promoting urban health and well-being. For example, the philanthropic foundation Fondation Botnar, committed to advancing the health and well-being of young people in cities across the globe, welcomes the agenda’s guiding principles and global research priorities. The Foundation seeks to create a just and equitable society based on universal values such as mutual aid, care, collaboration, democracy and human rights, and acknowledging agency of young people as a force for social change, in alignment with the urban health research agenda’s philosophy. 

Adopting a systems lens is at the heart of Fondation Botnar’s city engagements to address the complexities faced by our partners and target groups in their ambitions for positive change. Fondation Botnar’s global programmes such as OurCity Initiative, S^2^Cities (Safe and Sound Cities), Urban Futures, Healthy Cities for Adolescents and Youth Gamechanger Initiative^3^ all set out to tackle the social and environmental conditions in which people are born, grow, live, work and age, as these conditions shape and drive health outcomes. These initiatives aim to achieve their goals by building safe and liveable public spaces, creating livelihood opportunities, and improving nutrition through fostering sustainable urban food systems.

All the programmes have a context-based, localized response to needs and opportunities, and are committed to fostering young people’s civic engagement and right to participation in political processes and decision-making.

Such collective aspirations rely on building trust among stakeholders for triggering a process of co-producing of evidence on urban health needs that leads to co-designing action and fostering change. 

Possibly one of the biggest challenges of an urban health research agenda built on the paradigm shift of addressing determinants and drivers of health, and accounting for city systems’ transformation towards health and well-being of urban dwellers, is measuring the impact of interventions. The agenda’s research priorities and underlying principles call for a rethinking of impact measurement approaches, which are typically factor-based or linear and fall short of understanding complex, lived experiences and long-term social changes in cities.

For its programmes, Fondation Botnar, in collaboration with the Melbourne Centre for Cities, has developed a framework to gather evidence systematically and regularly from local multi-stakeholder action, and embedding learning in a project cycle to rethink determinants of success for multisectoral interventions in complex urban environments. The Evidence to Action framework^4^ strengthens links between research and practice by institutionalizing a process of collaborative data collection, learning and evidence building that informs Fondation Botnar’s engagement within and across diverse cities.

Fondation Botnar will advance co-production of knowledge grounded in community participation and collaboration, particularly with young people as relevant, but typically underrepresented stakeholders.

The agenda provides a valuable framework for an intensified focus on context-specific structures, mechanisms and dynamics, going well beyond the health sector. Uptake and implementation will be crucial, therefore calling for a proactive nurturing of collaborations, locally, regionally and globally, with new thinkers and actors, and genuine openness to embracing new approaches and tools for collective evidence generation and implementation. Philanthropic foundations, as much as a diversity of stakeholders from all sectors, including young people, should all have their equal say in a joint journey towards healthier societies in urban contexts.
